# Plasma branched-chain amino acids in chronic kidney disease: associations with atherogenic lipids and mortality

**DOI:** 10.1007/s00726-026-03511-7

**Published:** 2026-03-08

**Authors:** Mohamed E. Suliman, Abdul Rashid Qureshi, Dario Troise, Qianying Zhang, Peter Bárány, Olof Heimbürger, Peter Stenvinkel, Bengt Lindholm

**Affiliations:** 1 Renal Medicine, Department of Clinical Sciences, Intervention and Technology, Karolinska Institutet, M99 Karolinska University Hospital Huddinge, 14186 Stockholm, Sweden; 2 Nephrology, Dialysis and Transplantation Unit, Advanced Research Center on Kidney Aging (A.R.K.A.), Department of Medical and Surgical Sciences, University of Foggia, 71122 Foggia, Italy; 3 Department of Nephrology, Ruijin Hospital, Shanghai Jiao Tong University School of Medicine, Shanghai, China; 4 Renal Medicine, Department of Clinical Sciences, Intervention and Technology, Karolinska Institutet, M99 Karolinska University Hospital, 141 86 Stockholm, Sweden

**Keywords:** Branched-chain amino acids, Lipid profile, Inflammation, Chronic kidney disease, Mortality

## Abstract

Branched-chain amino acids (BCAAs) are essential nutrients that promote muscle protein anabolism but also associate with cardiometabolic diseases; however, their role in chronic kidney disease (CKD) remains unclear. We investigated plasma BCAA levels and their associations with metabolic parameters and survival in CKD patients. Plasma BCAA levels, along with clinical and laboratory parameters, were measured in 328 patients with CKD stage 5 (median age 54 years, 60% males). BCAA concentrations in 83 community-dwelling controls (median age 51 years, 66% males) served as comparators. Multivariate linear regression analysis was employed to identify predictors of BCAA levels. Competing risk regression analysis was conducted to assess 5-year risk of all-cause and cardiovascular mortality in relation to total and individual BCAAs (valine, isoleucine, and leucine). Plasma BCAA levels were lower compared to controls (*P* < 0.0001) and positively associated with triglycerides and the atherogenic index of plasma, while inversely associated with high density lipoprotein-cholesterol (HDL-C), Apo-A, and Lp (a). After adjustments for confounders, low vs. high tertile of total BCAAs associated with increased risk of cardiovascular mortality (sub-hazard ratio [sHR] 2.37, 95% confidence interval [CI], 1.08–5.21) and low tertile of valine associated with higher risk of both all-cause mortality (sHR 2.05, 95% CI 1.10–3.79) and cardiovascular mortality (sHR 2.46, 95% CI 1.15–5.26). In CKD, higher levels of BCAAs associated with an atherogenic lipid profile while lower BCAAs levels associated with increased cardiovascular mortality risk, and low valine was associated with higher both all-cause and cardiovascular mortality risk. Monitoring and potentially modulating BCAA levels could have prognostic or therapeutic implications in advanced CKD.

**Supplementary Information:**

The online version contains supplementary material available at 10.1007/s00726-026-03511-7.

## Introduction

Branched-chain amino acids (BCAAs), which include leucine (Leu), isoleucine (Ile), and valine (Val), are essential amino acids that play a crucial role in muscle protein synthesis and energy production. Beyond serving as substrates for protein synthesis, BCAAs act as signaling molecules, stimulating protein synthesis and regulating glucose and lipid metabolism, neurotransmitter and hormone synthesis, and mitochondrial biogenesis, and they have key roles for intestinal health and immunity (Nie et al. 2018, Holecek 2018).

On the other hand, high plasma BCAAs concentrations are closely associated with presence and severity of cardiometabolic alterations such as insulin resistance, metabolic syndrome, diabetes mellitus (DM), and obesity, as well as cardiovascular disease (CVD) (Batch et al. 2014). Adipose tissue, a dynamic organ essential for glucose and lipid metabolism, also influences BCAA balance through nutrient storage and lipolysis (Gannon et al. 2018). Moreover, associations between BCAAs and metabolic dyslipidemia have been reported and several studies have explored the connections between BCAAs and the lipid profile (Yang et al. 2016, Hamaya et al. 2021, Fukushima et al. 2019, Wang et al. 2019, Yamaguchi et al. 2017, Yamakado et al. 2015). Indeed, recent studies in the general population show that high levels of BCAAs are associated with elevated triglycerides (TG) and reduced high density lipoprotein-cholesterol (HDL-C) levels (Yang et al. 2016, Hamaya et al. 2021, Fukushima et al. 2019, Wang et al. 2019). Whereas the connection between BCAAs and the lipid profile is not completely clear, it has been suggested that it is partially mediated by insulin resistance, a common feature in individuals with elevated BCAAs levels, which associates with increased lipolysis and reduced clearance of circulating lipids, contributing to dyslipidemia (Newgard 2012). The finding of a significant association between BCAAs and insulin resistance suggests that BCAAs may play a major role in the modulation of insulin action (Lu et al. 2013). Circulating levels of the BCAAs are significantly higher in obese individuals compared to lean ones (Newgard et al. 2009) and BCAAs have been established as some of the most robust biomarkers for a range of cardiometabolic conditions, including obesity, insulin resistance, type 2 DM, and CVD (White et al. 2021). Furthermore, genetic variants linked to dyslipidemia and insulin resistance have been associated with increased concentrations of circulating BCAAs, suggesting that obesity and insulin resistance may drive the obesity-related rise in BCAAs (Wang et al. 2017).

Chronic kidney disease (CKD) patients often exhibit insulin resistance and dyslipidemia, along with disturbances in amino acid metabolism, particularly involving BCAAs (Cano et al. 2006). Patients with advanced CKD show decreased levels of plasma and intracellular BCAAs (Cano et al. 2006, Alvestrand et al. 1983, Bergström et al. 1990, Lindholm et al. 1989, Jones and Kopple 1978, Qureshi et al. 1998), likely due to impaired kidney amino acid metabolism and metabolic changes such as acidosis, and malnutrition (Cano et al. 2006, Kopple et al. 2005) and often present with elevated levels of TG and low-density lipoprotein cholesterol (LDL-C), decreased HDL-C levels, and altered lipoprotein composition.

Whereas links between BCAAs and lipoproteins have been reported in the general population, the existence of such a linkage in CKD patients has to the best of our knowledge not yet been explored. Therefore, in the present study, we evaluated associations between plasma BCAA concentrations and serum cholesterol, TG, HDL-C, and LDL-C, and estimated the risk of atherogenic dyslipidemia in relation to plasma BCAA levels, in patients with advanced CKD. We used the atherogenic index of plasma (AIP), reflecting the TG to HDL-C ratio, as a biomarker for atherogenic dyslipidemia (Niroumand et al. 2015). By considering two atherogenic lipid parameters, AIP provides insights into an individual’s risk of atherosclerosis.

While high plasma concentrations of BCAAs may be linked to cardiometabolic diseases, low circulating levels of essential nutrients such as BCAAs could potentially have a negative effect on survival. However, the association between BCAAs and mortality risk in CKD remains unclear and has not been conclusively determined. Therefore, we investigated these associations to gain a better understanding of possible links between plasma BCAA concentrations, atherogenic lipids, and clinical outcomes in CKD patients.

## Materials and methods

### Study populations

**Fig. 1:**
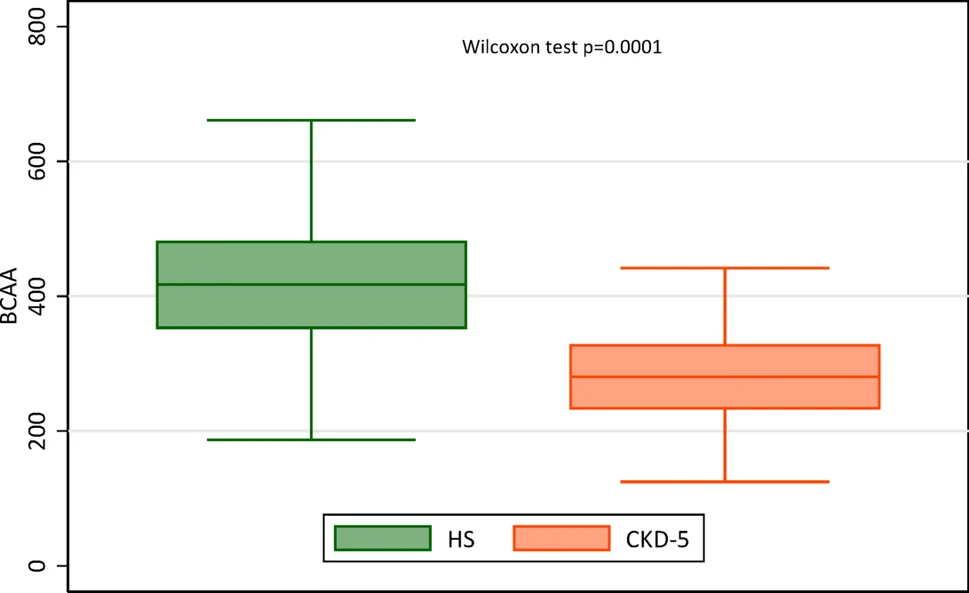
Box plots of plasma concentrations (mmol/L) of total branched-chain amino acids in 44 healthy subjects (HS) and 328 patients with CKD stage 5

This is a *post hoc*analysis of a cross-sectional study with follow-up data of mortality in a cohort of patients with CKD stage 5 (Stenvinkel et al. 1999) (FigureS1). The study conformed to and was conducted in accordance with the STROBE (Strengthening the Reporting of Observational Studies in Epidemiology) guidelines (Elm et al. 2014). The patients, who were recruited between December 1994 and April 2014, were investigated close to the start of dialysis treatment and were followed for up to five years. Exclusion criteria included age younger than 18 or older than 70 years, signs of overt infection, acute vasculitis, unwillingness to participate, and, for this study, missing BCAA measurement. Of 560 patients included in the cohort, 328 patients (198 (60%) males, median age 54 years, and median estimated glomerular filtration rate (eGFR) 6.3 ml/min per 1.73 m^2^) met the study criteria and were included in the current analyses, see Table 1. The causes of CKD were diabetic nephropathy (26%), hypertension and kidney vascular disease (21%), chronic glomerulonephritis (25%), and other causes (28%). Most patients (85%) were on antihypertensive medications, 59 (18%) patients were prescribed statins and other commonly used drugs in kidney failure such as erythropoietin, phosphate binders and potassium binders, and 61 (19%) patients received an oral amino acid supplementation.

**Table 1:** Baseline characteristics of all 328 CKD stage 5 patients and when divided into tertiles based on plasma concentration of total branched-chain amino acids (BCAAs)

Patient characteristics	Total	BCAAs First tertile	BCAAs Middle tertile	BCAAs Third tertile	*P*-value
Number	328	108	109	111	
Age, years	54 (43–63)	56 (47–63)	53 (44–63.)	52 (42–64)	0.67
Males, n (%)	198 (60%)	49 (45%)	69 (63%)	80 (72%)	< 0.001
DM, n (%)	104 (32%)	32 (30%)	37 (34%)	35 (32%)	0.79
CVD, n (%)	111 (34%)	38 (35%)	36 (33%)	37 (33%)	0.94
PEW, n (%)	103 (31%)	46 (44%)	32 (31%)	25 (24%)	0,007
BMI, kg/m^2^	24 (22–27)	24 (22–27)	24 (22–28)	24 (22–28)	0.74
eGFR, ml/min/1.73m^2^	6.4 (5.0–8.2.0.2)	6.3 (5.0–8.2.0.2)	6.6 (5.1–7.9)	6.5 (5.0–8.7.0.7)	0.60
CRP, mg/L	5.0 (2.0–15.0)	4.0 (1.6–15.0)	4.8 (2.0–15.0)	5.7 (2.6–14.0)	0.71
Hb, g/L	103 (94–114)	113 (93–115)	102 (94–113)	104 (96–112)	0.92
Glucose-related parameters					
HbA1c, %	5.1 (4.5–6.1)	5.0 (4.4–5.8)	5.1 (4.4–6.4)	5.1 (4.6–6.2)	0.83
Blood glucose, mmol/L	5.4 (4.8–6.6)	5.3 (4.7–5.9)	5.3 (4.8–7.4)	5.5 (5.0–7.3.0.3)	0.08
Plasma insulin, mU/L	16 (11–29)	14 (11–22)	16 (10–27)	18 (11–29)	0.20
HOMA-IR	3.9 (2.5–7.1)	3.3 (2.4–5.4)	4.7 (2.4–7.9)	4.4 (2.9–9.1)	0.02
Plasma lipid profile					
Triglycerides, mmol/L	1.9 (1.3–2.5)	1.8 (1.2–2.3)	1.9 (1.4–2.5)	2.0 (1.4–2.9)	0.23
Total cholesterol, mmol/L	5.2 (4.4–6.3)	5.5 (4.5–6.5)	5.3 (4.4–6.4)	4.9 (4.1–6.2)	0.21
HDL-C, mmol/L	1.2 (0.9–1.5)	1.4 (1.0–1.9.0.9)	1.1 (0.9–1.4)	1.1 (0.8–1.4)	< 0.001
LDL-C, mmol/L	3.1 (2.2–4.0.2.0)	3.0 (2.2–4.0.2.0)	3.2 (2.3–4.2)	2.9 (2.0–4.0)	0.31
AIP, ratio	0.4 (0.0–1.0)	0.2 (−0.2-0.7)	0.5 (0.0–1.0)	0.5 (0.1–1.2)	0.003
Lipoprotein(a), nmol/L	213 (91–499)	212 (85–596)	271 (113–562)	180 (64–381)	0.02
Apolipoprotein-A, g/L	1.3 (1.1–1.5)	1.4 (1.2–1.6)	1.2 (1.1–1.4)	1.2 (1.0–1.4.0.4)	< 0.001
Apolipoprotein-B, g/L	1.0 (0.8–1.3)	1.1 (0.8–1.2)	1.1 (0.8–1.3)	1.0 (0.8–1.3)	0.60
Plasma BCAAs					
Total BCAAs, µmol/L	281 (232–329)	219 (191–232)	281 (263–291)	356 (328–397)	< 0.001
Valine, µmol/L	152 (126–179)	112 (96–129)	152 (140–163)	195 (174–231)	< 0.001
Isoleucine, µmol/L	56 (43–68)	41 (35–50)	57 (49–65)	69 (58–85)	< 0.001
Leucine, µmol/L	67 (54–84)	54 (46–62)	72 (58–81)	88 (68–108)	< 0.001

Data presented as median (IQR, interquartile range)

*DM *Diabetes mellitus, *CVD* Cardiovascular disease, *PEW* Protein-energy wasting, *BMI* Body mass index, *eGFR* Estimated glomerular filtration rate, *CRP* C-reactive protein, *HbA1c* Glycated hemoglobin, *HOMA-IR* Homeostatic model assessment of insulin resistance, *HDL-C* High-density lipoprotein-cholesterol, *LDL-C* Low-density lipoprotein-cholesterol, *AIP* Atherogenic index of plasma, *BCAAs* Branched-chain amino acids

For comparative analyses, we used data on individual and total BCAAs of 83 community-dwelling control subjects (66% males, median age 51 (range 21–80) years) with similar age and sex distribution as the patients (Supplemental material, Table S1).

Informed written consent was obtained from each patient and control subject. The protocol of this study was approved by the Ethics Committee of the Karolinska Institute (EPN) at the Karolinska University Hospital Huddinge, Stockholm, Sweden. The study was conducted in adherence to the Declaration of Helsinki.

### Methods

After an overnight fast, venous blood samples were taken for analysis. Plasma and serum were separated and kept frozen at − 70^◦^C if not analyzed immediately. Plasma amino acids including BCAAs (Leu, Ile and Val) were measured by reversed-phase high-performance liquid chromatography (HPLC) with fluorometric detection as previously described (Suliman et al. 1996).

Serum cholesterol, HDL-C and TG were analyzed by standard enzymatic procedures (Boehringer Mannheim, Mannheim, Germany). LDL cholesterol (LDL-C) was calculated using the Friedewald formula (Friedewald et al. 1972), expressed in mmol/L as follows: LDL-C = total cholesterol − HDL-C − (triglycerides/2.2). Lipoprotein(a) was analyzed using a two-site immunoradiometric assay (Pharmacia, Uppsala, Sweden) and apolipoprotein-A and apolipoprotein-B were measured using an immunonephelometric procedure (Behring AG, Marburg, Germany). High-sensitivity C-reactive protein (hsCRP) was measured by the nephelometry method. ELISA commercial kits were used for measurements of interleukin (IL)−6, IL-10, IL-18, 8-hydroxy-2’-deoxyguanosine (8-OHdG), tumor necrosis factor alpha (TNF- α), soluble intercellular adhesion molecule-1 (sICAM-1) and soluble vascular cell adhesion molecule-1 (sVCAM-1). The remaining biochemical analyses were done using routine methods at Karolinska University Hospital at Huddinge. Glomerular filtration rate (GFR) was assessed in the CKD patients as an estimated GFR (eGFR) from serum creatinine levels using the 2021 CKD-EPI creatinine equation without race coefficient (Inker et al. 2021). Atherogenic index of plasma (AIP) was calculated as the logarithmically transformed ratio of the serum TG to high-density lipoprotein cholesterol (HDL-C) as described in (Dobiasova and Frohlich 2000):$\mathrm{A}\mathrm{I}\mathrm{P}=\mathrm{log}\frac{{TG}}{{HDL}-C}$. Homeostatic Model Assessment of Insulin Resistance (HOMA-IR) was calculated based on the following equation: Fasting insulin in µU/mL)×fasting glucose in mmol/L) **​/** 22.5. Body mass index (BMI) was calculated as the subject’s body weight in kilograms divided by the square of the subject’s height in meters (kg/m^2^). Subjective global assessment (SGA) of nutritional status was evaluated using the four-point SGA scale and protein energy wasting (PEW) was defined as SGA score > 1 (Qureshi et al. 1998).

### Outcome ascertainment

Survival among the 328 CKD stage 5 patients was measured from the day of examination until death or censoring for kidney transplantation or when they completed the 5-year follow-up period. No patients were lost to follow-up. The median follow-up period was 29.4 (range 0.9–60) months. Within the 5-year follow-up period, 82 patients (25%) died, of which 52 (63%) were cardiovascular deaths, and 166 patients (51%) underwent transplantation. Cardiovascular deaths were attributed to ischemic heart disease, heart failure, ischemic stroke, peripheral vascular disease, or hemorrhagic stroke.

### Statistical analysis

Data are expressed as number (percentage) or median (interquartile range, IQR). Statistical significance was set at the level of *P* < 0.05. Comparisons between two groups were assessed with the non-parametric Wilcoxon test for continuous variables and Chi-square test (*χ*^2^) for nominal variables. When comparing more than two groups we applied the non-parametric Kruskal-Walli’s test for continuous variables and *χ*^2^test for nominal variables. Non-parametric Spearman rank correlation analysis was used to determine associations between variables. Multivariate linear regression analysis with stepwise backward selection of variables was used to determine the predictors of total and individual BCAAs.

Restricted cubic spline curve analysis was performed to assess the associations between total BCAA and valine concentrations (as continuous variables) and the risk of all-cause mortality and cardiovascular mortality. Fine-Gray competing-risk regression analysis models with kidney transplantation as a competing risk were used to estimate the sub-distribution hazard ratio (sHR) with 95% confidence interval (95% CI) to quantify the association of total and individual BCAAs, expressed as tertiles, with mortality risk. Statistical analyses were performed using statistical software Stata Now 18.5 (Stata Corporation, College Station, TX, USA) and Statistical Analysis Systems (SAS) SAS 9.4 level 1 M8 (SAS Campus Drive, Cary, NC, USA).

## Results

### Characteristics of the CKD patients

A total of 328 CKD patients (198 males and 130 females) with a median (IQR) age of 54 (43–63) years were included in the study (Table 1). Their median eGFR was 6.3 (4.9–7.9) ml/min/1.73 m^2^, 104 (32%) had DM and 111 (34%) had CVD. Moreover, Table 1 shows glucose-related parameters, plasma lipid profile including AIP, and total and individual BCAAs, for all patients and when patients are divided into tertiles of total BCAAs.

### BCAAs in patients and controls

Plasma concentrations of total (T-BCAA) and individual BCAAs were significantly lower in CKD patients compared to community-dwelling controls (Fig. 1 and Table S1), except for Ile, for which the difference did not reach statistical significance.

### BCAAs in different CKD patient groups

The plasma concentrations of T-BCAA (Fig. S2) and the three individual BCAAs (Table S2) were higher (all *p* < 0.0001) in male patients compared to female patients. Similarly, plasma concentrations of total BCAAs (T-BCAA) and the three individual BCAAs were significantly higher in male compared to female control subjects (Fig. S3). However, the plasma BCAA concentrations did not differ significantly between CKD patients with and without DM, CVD, and statin treatment, respectively (Fig. S2). Moreover, the concentrations of BCAAs did not differ significantly between 61 patients (19%) who received amino acid supplementation and those who did not (Table S3).

### Associations of BCAAs with lipid profile

The individual BCAAs were strongly correlated with each other and with lipid components (Table 2). TG was positively and significantly correlated with T-BCAA and Val. Moreover, HDL-C was negatively and significantly correlated with T-BCAA, Val, Ile, and Leu. Similarly, AIP, Lp(a), and Apo-A were negatively and significantly correlated with T-BCAA, Val, and Ile, but not with Leu. Serum cholesterol, LDL-C, and Apo-B did not show significant associations with T-BCAA or individual BCAAs.

**Table 2:** Spearman rank correlation matrix of branched chain amino acids and lipid profile in 328 CKD stage 5 patients

	BCAAs	Val	Ile	Leu	TG	HDL	AIP	Chol	LDL	Lp(a)	Apo-A
Val	0.93^d^										
Ile	0.73^d^	0.59^d^									
Leu	0.63^d^	0.40^d^	0.29^d^								
TG	0.13^a^	0.15^b^	0.08	0.02							
HDL	−0.26^d^	−0.24^d^	−0.24^d^	−0.15^b^	−0.45^d^						
AIP	0.22^c^	0.22^c^	0.17^b^	0.10	0.89^d^	−0.79^d^					
Chol	−0.08	−0.05	−0.05	−0.10	0.41^d^	0.11	0.22^c^				
LDL	−0.02	−0.01	0.02	−0.05	0.29^d^	−0.09	0.23^c^	0.91^d^			
Lp(a)	−0.12^a^	−0.13^a^	−0.10	−0.05	−0.08	0.15^b^	−0.13^a^	0.12^a^	0.11		
Apo-A	−0.23^c^	−0.24^d^	−0.21^c^	−0.07	−0.25^d^	0.75^d^	−0.53^d^	0.26^d^	0.09	0.12^a^	
Apo-B	0.02	0.01	0.04	−0.02	0.46^d^	−0.18^b^	0.39^d^	0.84^d^	0.87^d^	0.12^a^	0.08

*BCAAs* Branched-chain amino acids, *Val* Valine, *Ile* Isoleucine, *Leu* Leucine, *TG* Triglycerides, *HDL* High-density lipoprotein-cholesterol, *AIP* Atherogenic index of plasma, *Chol* Cholesterol, *LDL* Low-density lipoprotein-cholesterol, *Lp(a)* Lipoprotein (a), *Apo-A* Apolipoprotein A, *Apo-B*, Apolipoprotein B

Significance levels: a < 0.05, b < 0.01, c < 0.001 and d < 0.0001

**Fig. 2:**
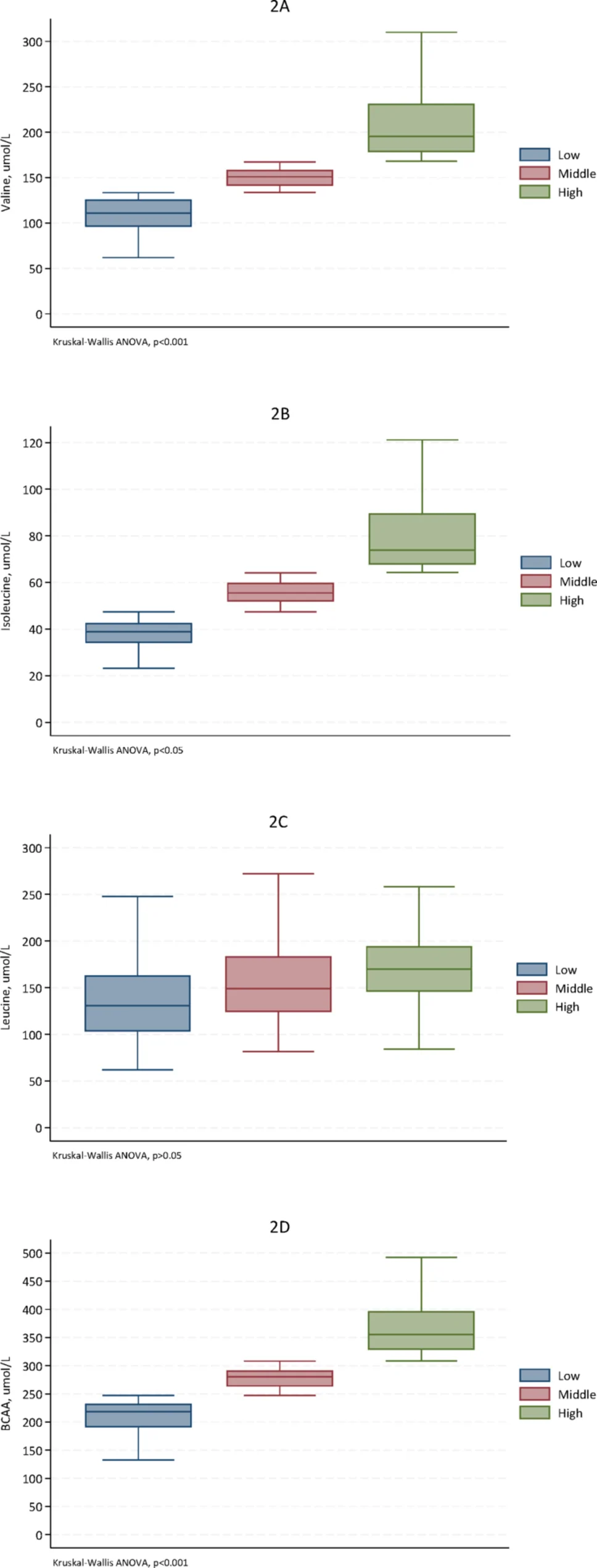
Box plots of the concentrations (μmol/L) of individual amino acids: valine (**2A**), isoleucine (**2B**), leucine (**2C**) and the sum of branched-chain amino acids (BCAA, **2D**) for each tertile of atherogenic index of plasma (AIP) of 328 CKD stage 5 patients. The median (interquartile range) AIP ratio for the low, middle and high tertiles were -0.262 (-0.642-0.000), 0.379 (0.223-0.565) and 1.220 (0.963-1.584), respectively

To further explore links between AIP and BCAAs, we divided the patients into AIP tertiles and compared the concentration of T-BCAA and individual BCAAs between the AIP tertiles. The high AIP tertile had significantly higher levels of T-BCAA, Val and Ile, but not Leu, compared to the low tertile (Fig. 2).

The association between BCAAs and atherogenic lipids was further investigated by multivariate linear regression analysis to determine variables independently associated with BCAAs (Table 3). In a model including age, sex, DM, CVD, BMI, AIP and plasma insulin, higher AIP, higher plasma insulin and male sex emerged as independent predictors of plasma T-BCAA concentration, whereas age, DM and BMI were not significantly associated with T-BCAA.

**Table 3:** Predictors of 1-standard deviation (SD) increase of plasma concentrations of total (T-BCAAs) and individual branched-chain amino acids in 328 CKD 5 patients

	T-BCAAs		Valine		Isoleucine		Leucine	
	Beta	*P*-value	Beta	*P*-value	Beta	*P*-value	Beta	*P*-value
1-SD of age, years	−0.012	ns	−0.007	ns	−0.019	ns	−0.008	ns
Sex, F vs. M	−0.258	0.0001	−0.215	0.0001	−0.253	0.0001	0.163	0.004
Presence of DM	0.021	ns	−0.020	ns	0.010	ns	0.090	ns
Presence of CVD	−0.067	ns	−0.075	ns	−0.102	ns	0.189	ns
1-SD of BMI	0.050	ns	0.041	ns	0.003	ns	0.070	ns
1-SD of AIP	0.153	0.006	0.177	0.002	0.120	0.03	0.031	ns
1-SD of plasma insulin	0.127	0.02	0.099	0.07	0.097	0.07	0.113	0.04

Multivariate linear regression analysis with stepwise backward selection of variables

The model including sex, age, diabetes mellitus (DM), cardiovascular disease (CVD), body mass index (BMI), plasma insulin and atherogenic index of plasma (AIP). AIP is a ratio of the log of TG to HDL-C

*ns* Non-significant

### Associations of BCAA with glucose-related factors and inflammatory biomarkers

As shown in Table 4, blood glucose, plasma insulin and HOMA-IR were positively and significantly correlated with T-BCAA and Val, and HbA1c was inversely and significantly correlated with Leu.

**Table 4:** Correlations between plasma concentrations of total (T-BCAA) and individual BCAAs with glucose-related parameters, estimated glomerular filtration rate (eGFR) and inflammatory markers in 328 CKD 5 patients

	T-BCAA	Val	Ile	Leu
HbA1c	0.10	0.06	0.03	0.17^***^
Glucose	0.12^*^	0.12^*^	0.06	0.06
Insulin	0.12^*^	0.14^**^	0.06	0.02
HOMA-IR	0.17^**^	0.20^***^	0.10	0.05
eGFR	0.15^**^	0.18^***^	0.08	0.02
TNF-α	−0.15^**^	−0.14^**^	−0.18^***^	0.01
WBC	0.04	0.01	0.04	0.08
hsCRP	0.05	0.05	0.06	−0.01
IL-6	−0.01	−0.04	−0.002	0.04
IL-10	−0.05	−0.05	−0.03	−0.07
IL-18	0.06	0.10	0.03	−0.02
sICAM-1	−0.05	−0.03	−0.11	−0.02
sVCAM-1	−0.04	−0.05	0.02	0.01
8-OHdG	−0.01	−0.02	−0.05	0.01

Spearman rank correlation (ρ)

Significance levels: *<0.05, **<0.01 and ***<0.001

*HbA1c* Hemoglobin A1c, *HOMA-IR* Homeostatic model assessment for insulin resistance, *eGFR* Estimated glomerular filtration rate, *TNF- α* Tumor necrosis factor-alpha, *WBC* White blood cells, *hsCRP* High-sensitive C-reactive protein, *IL−6, IL-10, IL-18* Interleukin, *sICAM-1* Soluble intercellular adhesion molecule-1, *sVCAM-1* Soluble vascular cell adhesion molecule-1, *8-OHdG* 8-hydroxy-2’-deoxyguanosine

TNF-α correlated inversely with T-BCAA, Val, and Leu (Table 4). Other inflammatory markers such as WBC, hsCRP, IL-6, IL-10, IL-18, sICAM-1, sVCAM-1, and 8-OHdG were not significantly correlated with the concentrations of BCAAs.

### Associations of BCAA with nutritional status

Based on SGA, 111 (34%) of the CKD patients were classified as having PEW. The patients with PEW exhibited significantly lower total BCAA levels compared with well-nourished CKD patients (254 [224–305] µmol/L vs. 290 [237–334] µmol/L, *P* = 0.002) (Fig. S4). In addition, TNF-α concentrations were significantly higher in patients with PEW than in those with normal nutritional status (11.1 [8.5–15.2] pg/mL vs. 9.8 [7.9–12.5] pg/mL, *P* = 0.03) Fig. S5.

### BCAAs and mortality

**Fig. 3:**
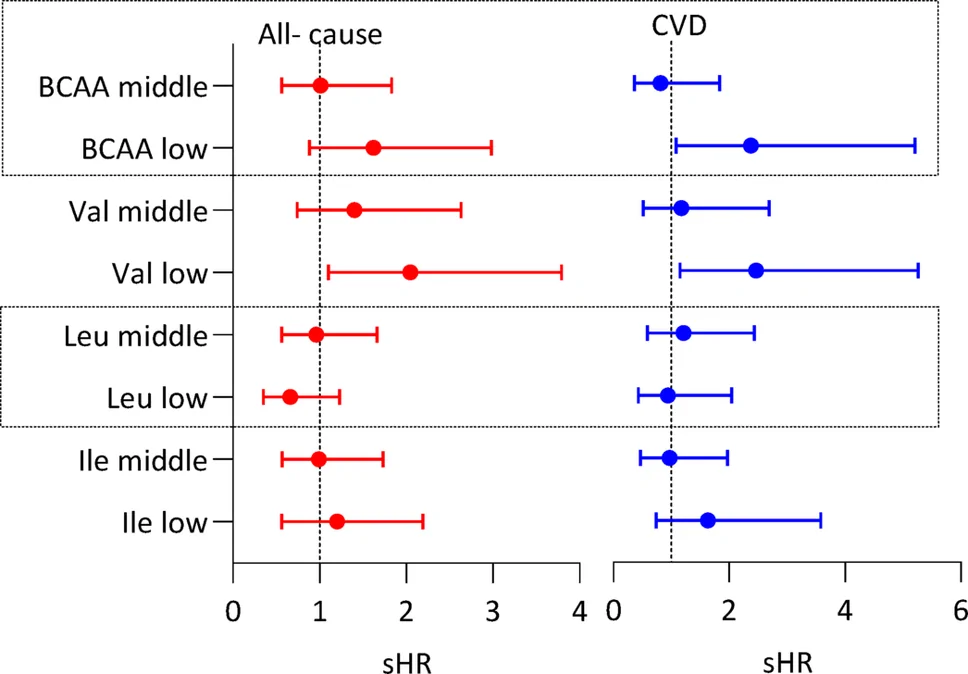
Competing-risk regression analysis models for cardiovascular and all-cause mortality risk based on tertiles of total BCAA, valine (Val), leucine (Leu) and isoleucine (Ile), respectively, among 328 CKD stage 5 patients during a follow-up period of up to five years. Values are estimated sub-hazard ratio (sHR) with 95% confidence interval. The high tertile served as the reference group. Each model was adjusted for age, sex, diabetes mellitus, cardiovascular diseases, estimated glomerular filtration rate, body mass index, serum albumin, atherogenic index of plasma (AIP) and plasma insulin

The associations of low and middle tertiles vs. high tertile of T-BCAA and individual BCAAs with cardiovascular and all-cause mortality were investigated using competing-risk regression analysis models, adjusted for age, sex, DM, CVD, eGFR, BMI, AIP, serum albumin and plasma insulin (Fig. 3 and Table S4). The low T-BCAA tertile was associated with increased risk of cardiovascular mortality (sub-hazard ratio [sHR] 2.37, 95% confidence interval [CI], 1.08–5.21) and the low tertile of valine was associated with higher risk of both all-cause mortality (sHR 2.05, 95% CI 1.10–3.79) and cardiovascular mortality (sHR 2.46, 95% CI 1.15–5.26).

**Fig. 4:**
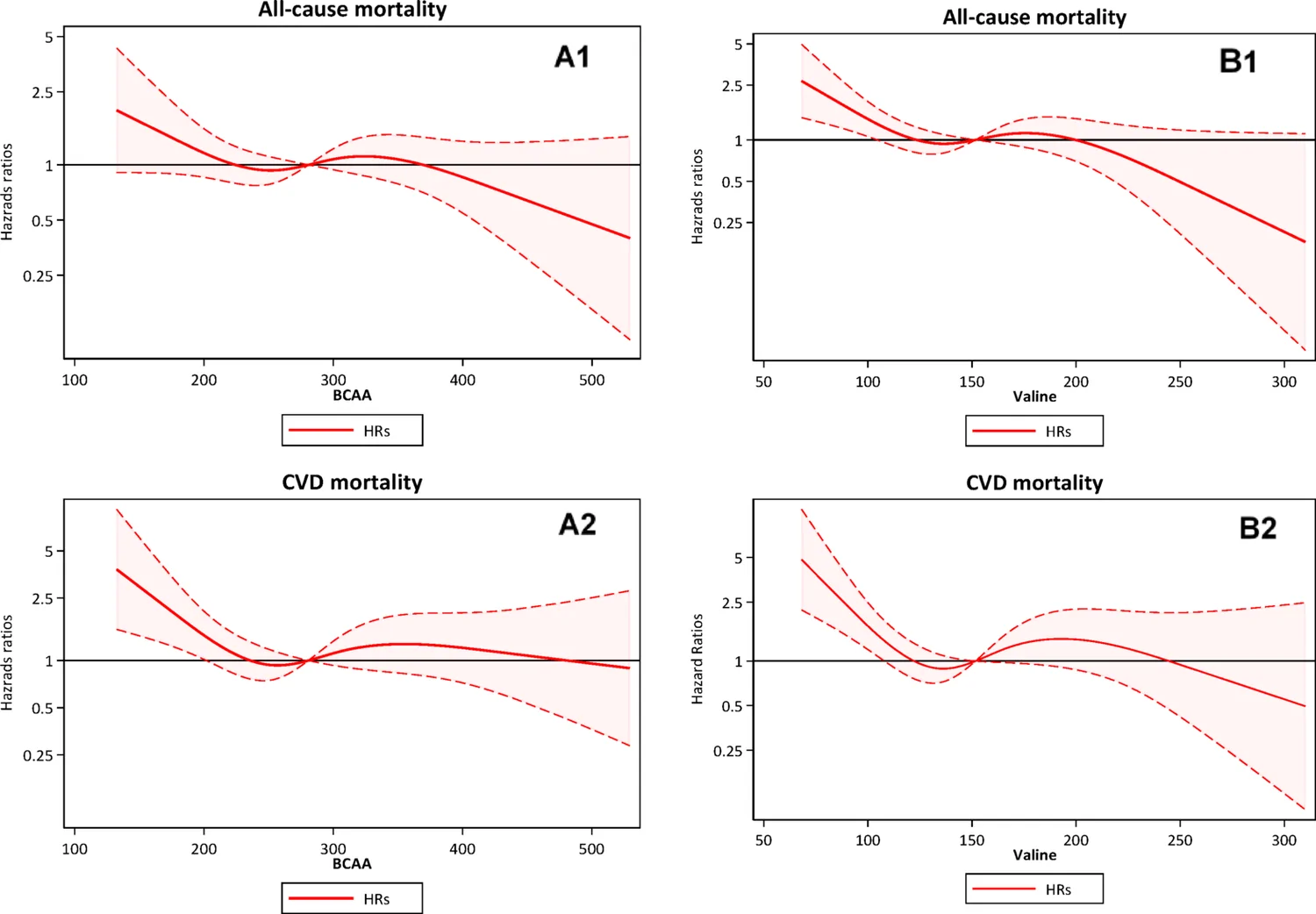
Restricted cubic spline curves showing the relationship between baseline plasma concentrations (mmol/L) of total branched chain amino acids (BCAA) (**A1** and **A2**) and valine (**B1** and **B2**), as continuous variables, and the subsequent 5-year all-cause and cardiovascular mortality risk, expressed as HRs and 95% CI in 326 CKD stage 5 patients

Restricted cubic spline curve analysis showed that lower plasma concentrations of T-BCAA and Val, as continuous variables, were associated with increased 5-year all-cause and cardiovascular mortality risk (Fig. 4).

## Discussion

Our study provides several important insights into the complex relationship between BCAAs, atherogenic lipids and mortality risk in patients with advanced CKD. Their plasma concentrations of BCAAs, which were lower than in community-dwelling controls, were inversely associated with increased cardiovascular mortality, and lower Val concentration was associated with increased both all-cause and cardiovascular mortality risk. Finally, higher plasma BCAA levels were positively associated with TG and AIP, and inversely associated with HDL-C, Apo-A, and Lp(a).

### BCAAs in different CKD groups

During CKD, abnormalities in BCAA metabolism occur due to the reduced kidney contribution to amino acid metabolism, compounded by declining kidney function and the accumulation of uremic toxins, which disrupt whole body nitrogen metabolism. Previous studies have demonstrated that patients with advanced CKD, including those undergoing hemodialysis (HD) or peritoneal dialysis, exhibit reduced levels of essential amino acids, including BCAAs (Bergström et al. 1990, Lindholm et al. 1989, Qureshi et al. 1998, Ikeda 2020). In our study, BCAA levels were significantly lower in the CKD patients compared to a group of community-dwelling controls. The precise cause of lower BCAA levels in CKD remains unclear.

In CKD stage 5, reduced circulating BCAA levels likely reflect a combination of dietary and disease-related factors that are difficult to fully disentangle. Although dietary protein restriction and reduced appetite may contribute to lower BCAA intake in advanced CKD, profound metabolic disturbances, including metabolic acidosis, insulin resistance, uremic toxin accumulation, chronic inflammation, and accelerated muscle proteolysis, are key drivers of altered amino acid metabolism independent of intake (Cano et al. 2006). However, we acknowledge that in the absence of direct measurements of dietary protein intake (e.g., food records) or objective markers such as normalized protein catabolic rate (nPCR), we cannot exclude the possibility that reduced protein intake substantially contributed to the lower circulating BCAA levels observed in our cohort. Therefore, the decline in BCAAs across CKD stages and their association with mortality may, at least in part, reflect the severity of dietary protein restriction rather than a distinct metabolic or inflammatory phenotype. In support of metabolic contribution, BCAA concentrations did not differ between patients receiving oral amino acid supplementation and those who did not (Table S3), and lower BCAA levels were associated with protein-energy wasting (Fig. S4) and higher TNF-α concentrations (Fig. S5). Moreover, the associations between low total BCAA and valine concentrations and increased mortality risk (Fig. 3) remained significant after adjustment for serum albumin, BMI, and other major confounders, indicating that low circulating BCAA levels may serve as integrative biomarkers of disease severity, reflecting a combination of nutritional status, metabolic stress, and catabolic burden in advanced CKD. Nevertheless, albumin and BMI are imperfect surrogates for dietary intake and muscle mass, and residual confounding by nutritional status cannot be excluded. Importantly, we did not have direct measurements of muscle mass or body composition, which are likely to influence circulating BCAA concentrations. Loss of skeletal muscle mass, common in advanced CKD, could directly reduce the systemic BCAA pool and thereby confound associations with inflammation and mortality.

Previous studies have shown a stepwise decline in circulating total and individual BCAA concentrations which become progressively more evident with declining kidney function, with relatively modest or inconsistent changes in early CKD stages but marked reductions in advanced CKD and dialysis populations (Cano et al. 2006, Alvestrand et al. 1983, Bergström et al. 1990, Lindholm et al. 1989, Jones and Kopple 1978, Qureshi et al. 1998, Ikeda 2020).

Our findings also revealed strong intercorrelations among plasma concentrations of BCAAs in CKD patients, consistent with findings by Ikeda et al. in both HD patients and healthy older individuals and by Luo et al. (2023) in patients with hypertension-attributed CKD. These observations support previous explanations that attributed the strong correlation among BCAAs to shared metabolic enzymes (Brosnan and Brosnan 2006).

Furthermore, in CKD patients and in community-dwelling controls, BCAA concentrations were significantly higher in males compared to females, consistent with previous findings in HD patients and healthy individuals (Ikeda 2020). The higher levels of BCAAs in males may be attributed to differences in muscle mass, hormone status, and body fat distribution, all of which affect amino acid metabolism differently between sexes.

### Associations BCAAs with lipid profile

A novel and intriguing finding in our study is the specific correlation between BCAA levels and lipid profiles. To our knowledge, this is the first study to examine the association between plasma BCAA concentrations and the circulating lipid profile in CKD patients. Although BCAA levels in our cohort were lower than those typically observed in healthy individuals, they were still positively associated with atherogenic lipids, as indicated by the Atherogenic Index of Plasma (AIP), a composite biomarker derived from the ratio of TG to HDL-C. Consistent with previous studies in non-kidney disease populations (Yang et al. 2016, Hamaya et al. 2021, Fukushima et al. 2019, Wang et al. 2019), we found that BCAA concentrations were significantly positively correlated with TG and AIP, and negatively correlated with HDL-C, Apo-A, and Lp (a). Interestingly, no significant correlation was observed between BCAA levels and total cholesterol or LDL-C concentrations.

BCAAs are crucial in protein synthesis and energy metabolism and have been shown to regulate both glucose and lipid metabolism (Nie et al. 2018). The interaction between BCAAs and lipids involves complex metabolic pathways, where BCAAs influence enzymes and signalling pathways critical for lipid synthesis and breakdown (Nie et al. 2018, Choi et al. 2024). Although BCAAs are primarily metabolized in skeletal muscle, their catabolism is linked to the production of acyl-CoA intermediates that can enter lipid synthesis pathways (Zhang et al. 2017), potentially leading to increased production of specific lipids. Low BCAA levels, as in CKD, may impair the regulation of these pathways, resulting in lipid accumulation in the bloodstream. Additionally, BCAAs may influence lipid transport by affecting lipoprotein levels, including those responsible for carrying cholesterol and triglycerides (Galli et al. 2023). This interaction may contribute to dyslipidemia, further highlighting the complex role of BCAAs in lipid metabolism and cardiovascular risk. However, given the cross-sectional design and the lack of dietary and body composition data, these associations should be interpreted as metabolic correlations rather than evidence of causality. It is possible that shared upstream determinants—such as nutritional status, muscle mass, insulin resistance, or CKD-related metabolic alterations—contribute to both BCAA concentrations and lipid abnormalities. In CKD patients, dysregulated lipid metabolism and reduced circulating BCAA levels may coexist as markers of heightened cardiovascular risk. This association may reflect interconnected mechanisms, including the promotion of atherogenesis, enhanced insulin resistance, chronic inflammation, oxidative stress, muscle wasting, and endothelial dysfunction (Gai et al. 2019). However, our data do not allow us to determine whether BCAAs function as mediators, markers, or merely bystanders within this complex network of metabolic disturbances.

### Associations of BCAAs with glucose-related factors

Many studies have reported a close association between elevated BCAA levels and the development of insulin resistance, DM, and hypertension (Cuomo et al. 2022, Alqudah et al. 2024, Bloomgarden 2018). However, in our study, BCAA concentrations in CKD patients with DM or hypertension did not significantly differ from those without these conditions. Given that insulin resistance is a common and early feature of kidney failure, independant of diabetes status, this lack of group difference is not unexpected.

Despite this, we observed significant associations between T-BCAA and individual BCAA levels with blood glucose and plasma insulin concentrations, suggesting a link with glucose regulation. Moreover, the HOMA-IR was positively and significantly correlated with T-BCAA and val concentrations. There was also a positive trend between BCAAs and HbA1c; however, this reached statistical significance only for Leu (Table 4). These findings support a relationship between BCAAs and glucose-related traits in advanced CKD, although interpretation requires caution. Importantly, HbA1c may underestimate glycemic status in kidney failure due to shortened red blood cell lifespan and altered erythropoiesis, limiting its reliability in this population. Therefore, associations between BCAAs and HbA1c should be interpreted carefully.

The physiological relationship of circulating BCAAs and insulin dynamics is complex and context dependent. Circulating BCAA concentrations typically decline following insulin secretion, such as after an oral glucose tolerance test (OGTT), and reduced levels have been described in certain stages of mild or early insulin resistance. Consequently, the direction and magnitude of associations between BCAAs and insulin resistance may vary according to metabolic context. Notably, studies by Newgard et al. (2012, 2009) (White et al. 2021) demonstrated that elevated circulating BCAA levels are strongly associated with insulin resistance and type 2 diabetes in individuals without kidney disease. Their findings suggest a contributory role of BCAAs in the pathogenesis of metabolic disease in those settings. In contrast, advanced CKD represents a fundamentally different metabolic milieu, characterized by reduced renal clearance, altered amino acid handling, muscle wasting, chronic catabolic stress, and often dietary protein restrictions. Under such conditions, regulation of BCAA metabolism may differ substantially from that observed in obesity or type 2 diabetes without kidney disease.

Taken together, our findings indicate that circulating BCAA levels are associated with glucose-related variables in advanced CKD. However, these relationships should be viewed as situational metabolic associations rather than evidence of causality. In the absence of direct assessments of dietary intake and muscle mass, it remains unclear whether BCAAs actively contribute to glucose dysregulation or instead reflect nutritional status, muscle turnover, or disease severity. Longitudinal and mechanistic studies are needed to clarify the role of BCAAs in glucose homeostasis in CKD and to determine how these pathways differ from those described in non-CKD populations.

### Associations of BCAAs with inflammatory markers

Our study did not find significant associations between total or individual BCAAs and inflammatory markers such as CRP or IL-6. However, there was a significant negative association between TNF-α and total BCAAs, Val, and Ile, but not Leu. Inflammatory cytokines like TNF-α and IL-6 modulate BCAA metabolism and are key mediators of muscle protein breakdown, altering circulating amino acid levels, including BCAAs (Sandri 2008). TNF-α, in particular, promotes muscle protein catabolism via the ubiquitin-proteasome pathway, contributing to elevated BCAA levels and systemic metabolic disturbances (Sandri 2008). TNF-α, IL-1β, and IL-6 are central pro-inflammatory cytokines that drive myofibrillar protein degradation, inhibit protein synthesis, and lead to muscle atrophy. TNF-α also stimulates reactive oxygen species (ROS) production via the mitochondrial electron transport chain, activating NF-κB, which further enhances the ubiquitin-proteasome system, accelerating muscle protein breakdown and atrophy (Wang et al. 2014). TNF-α is produced by various cells, including macrophages, lymphocytes, and skeletal muscle cells, and contributes to local and systemic inflammation upon binding to its receptors (Zhou et al. 2016). In CKD patients and those on hemodialysis, persistent activation of innate and adaptive immunity results in “inflammaging,” a state of chronic low-grade inflammation (Jofre et al. 2006, Hernandez-Segura et al. 2018). Despite these mechanistic links, the inverse association between TNF-α and BCAAs observed in our study should be interpreted with caution. In non-CKD populations, inflammation and insulin resistance have often been associated with elevated rather than reduced BCAA levels. Therefore, it would be unjustified to conclude that inflammation per se directly causes reduced BCAA concentrations in CKD. A more parsimonious explanation is that inflammation contributes to anorexia, reduced dietary intake, and muscle wasting, which in turn lower circulating BCAA levels. In the absence of detailed dietary assessments and direct measurements of muscle mass, these pathways cannot be disentangled. Accordingly, the observed inverse association between TNF-α and BCAAs likely reflects a situational relationship within the CKD context rather than a direct causal effect of inflammation on BCAA reduction. Our findings are more consistent with the interpretation that lower BCAA concentrations reflect poorer nutritional and muscle status, which may coexist with higher inflammatory burden. This interpretation is supported by our observation that malnourished patients had significantly higher TNF-α levels than well-nourished patients.

At the same time, experimental data suggest that BCAAs may themselves enhance inflammatory signaling by activating NF-κB in immune cells, thereby increasing IL-6 and TNF-α production (Zhenyukh et al. 2017). Thus, TNF-α may promote muscle wasting and altered amino acid metabolism, while BCAAs may, under certain conditions, amplify inflammatory responses. Nevertheless, given the lack of consistent associations with other inflammatory markers in our study, the overall pattern more strongly supports a nutritional or catabolic explanation. In advanced kidney failure, reduced circulating BCAA levels are therefore more plausibly interpreted as indicators of compromised nutritional and muscle status rather than direct consequences of systemic inflammation.

### BCAAs and mortality

In our study, we observed that individuals in the low tertile of Val level, compared to those in the high tertile, had an increased risk of cardiovascular and all-cause mortality. Additionally, lower levels of total BCAAs were linked to higher cardiovascular mortality risk but not to all-cause mortality risk. While in our study, no significant associations were found between Ile or Leu and neither cardiovascular nor all-cause mortality, a recent study by Luo et al. (2023) reported that in hypertensive individuals with advanced non-diabetic CKD, higher levels of Val, Leu, and Ile were associated with reduced cardiovascular mortality, with higher levels of Leu and Ile, but not Val, also being linked to lower all-cause mortality.

Previous studies in non-kidney disease populations have yielded inconsistent results. For instance, a meta-analysis by Teymoori et al. (2023), which included nine cohort studies and one case–control study, found no association between total or individual BCAAs and the risk of all-cause mortality. On the other hand, Fung et al. (2023)demonstrated a U-shaped relationship between BCAA levels and mortality in older individuals with hypertension and/or diabetes mellitus, with both high and low BCAA levels being associated with increased mortality. In contrast, among individuals without these conditions (Fung et al. 2023), higher BCAA levels were associated with improved survival. Moreover, the LURIC study (Moissl et al. 2023) found that elevated BCAA levels in a Caucasian population were associated with a reduced long-term risk of both all-cause and cardiovascular mortality, with this relationship persisting regardless of obesity and/or diabetes status. The study by Deelen et al. (2019)further supported these findings, showing that higher levels of Val and Ile were associated with decreased mortality. Moreover, in a recent study Li et al. (2025) reported that lower total BCAA levels were associated with increased all-cause mortality in patients with liver cirrhosis.

Importantly, given the lack of adjustment for direct measures of dietary protein intake and muscle mass, the associations between lower BCAA levels and mortality observed in our study should not be interpreted as evidence of a causal role of BCAA deficiency. Rather, low BCAA concentrations may represent an integrated marker of disease severity, nutritional compromise, muscle wasting, or overall catabolic burden. Thus, while lower BCAA levels were independently associated with mortality after multivariable adjustment, residual confounding by unmeasured factors, particularly protein intake and body composition, cannot be excluded.

### Strengths and limitations

Our study is, so far, the most comprehensive exploration of the relationship between BCAAs, lipid profiles, and mortality risk among individuals with advanced CKD, with a relatively large sample size, detailed phenotyping and inclusion of age- and sex-matched controls for comparative analysis of BCAA concentrations. Additionally, no patients were lost to follow-up. However, some limitations should be considered when interpreting our findings. First, as this is an observational study, causality cannot be established. Second, BCAAs were measured only at the study’s outset. Third, detailed data on dietary protein intake or objective estimates such as normalized protein catabolic rate (nPCR) were not available in this cohort. Consequently, we were unable to adequately adjust for the potential confounding effect of dietary protein restriction, and residual confounding by differences in protein and BCAA intake cannot be excluded. This limitation is particularly important given that dietary restriction is common in advanced CKD and may substantially influence circulating BCAA concentrations. Fourth, we did not have direct measurements of skeletal muscle mass or body composition. Because skeletal muscle is a primary site of BCAA metabolism and a major determinant of circulating amino acid pools, the absence of body composition data limits our ability to disentangle the effects of muscle wasting from metabolic or inflammatory influences. Taken together, the lack of precise assessments of both dietary protein intake and muscle mass represents a significant limitation and precludes causal interpretation of the observed associations. Finally, unmeasured confounders may also have influenced the results, despite our efforts to account for potential confounders such as age, sex, BMI, DM, CVD, eGFR, plasma insulin, atherogenic lipids, and inflammation.

## Summary and conclusion

In patients with CKD stage 5, plasma concentrations of total and individual BCAAs were lower than in age- and sex matched healthy controls, lower in females than in males, and inversely correlated with TNF-α. Whereas higher levels of BCAAs were associated with an atherogenic lipid profile including higher AIP and glucose-related factors including HOMA-IR, competing risk regression analysis showed that lower levels of total BCAAs and valine were associated with increased cardiovascular mortality risk and lower plasma valine concentration was associated also with higher all-cause mortality risk.

These findings provide insights into the intricate relationships between BCAA levels, lipid profiles, glucose-related compounds, inflammatory parameters, and mortality in CKD patients. However, given the observational design and the lack of detailed dietary intake data and direct measurements of muscle mass, the results should be interpreted as evidence of clinically relevant associations rather than proof of causality. In particular, we cannot exclude the possibility that reduced protein intake, dietary restrictions, or sarcopenia substantially contributed to lower circulating BCAA levels and their association with adverse outcomes. Taken together, BCAAs may represent an integrated biomarker of nutritional and metabolic status in advanced CKD rather than a direct mechanistic driver of inflammation or mortality. The underlying pathways are likely multifactorial and remain to be fully elucidated. Further research, particularly longitudinal studies and interventional trials, is needed to clarify the role of BCAAs in CKD, their relationship to metabolic disturbances and disease progression, and their potential impact on patient outcomes. A deeper understanding of these mechanisms may ultimately contribute to the development of novel strategies aimed at improving prognosis in patients with advanced CKD.

## Supplementary Information

Below is the link to the electronic supplementary material.

Supplementary Material 1

## Data Availability

Data described in the manuscript, code book, and analytic code will be made available upon reasonable request pending application to the corresponding author.
