# Supplementary material for: Plasma branched-chain amino acids in chronic kidney disease: associations with atherogenic lipids and mortality
Source: Amino Acids. 2026 Mar 8;58(1):20. doi: 10.1007/s00726-026-03511-7 (PMC12979348; doi:10.1007/s00726-026-03511-7)
Supplement: Supplementary file 1 — Supplementary Material 1 [file 726_2026_3511_MOESM1_ESM.docx]

**Supplementary material**

Median plasma concentrations of total branched-chain amino acids (T-BCAA) and individual branched-chain amino acids were significantly lower in CKD stage 5 patients than in 83 community-dwelling control subjects (median age 51 (range 21-80) years, 55 males) with similar age and sex distribution as the patients (**Table S1**).

For comparative analyses, we used data on BCAAs and AAAs from 83 community-dwelling control subjects (median age 51 (range 21-80) years, 55 males) with similar age and sex distribution as the patients. The selection and clinical characteristics of the control subjects are described in (30, 31) (Mukai H et al PMID: 29702682, and Ghanavatian S et al PMID: 23567479).

The T-BCAA concentrations in CKD patients with and without DM (280 {234 - 334} vs. 281 {232 - 328} μmol/L), CVD (264 {231 – 338}μmol/L vs. 286 {232 – 327}μmol/L) and statin medication (271 {231 – 323) μmol/L vs. 284 {232 - 332} μmol/L), were not significantly different (**Fig. S2**). Likewise, the concentrations of individual BCAAs, Val, Ile, and Leu, also did not differ significantly between patients with these conditions and their counterparts (**Table S2**).

On the other hand, plasma concentrations of T-BCAA (Fig. S2) and individual BCAAs were significantly higher in male patients compared to female patients: T-BCAA 292 {247- 367}μmol/L vs. 259 {219 - 305}μmol/L; Val 158 {130 - 191}μmol/L vs. 142 {111 – 165}μmol/L; Ile 60 {50 – 72}μmol/L vs. 47 {39 - 61}μmol/L; Leu 71 {58 – 88}μmol/L vs. 62 {49 – 79}μmol/L, all with p-values <0.0001), respectively. Similarly, the plasma concentration of T-BCAA and individual BCAAs were significantly higher in male compared to female healthy subjects (**Fig. S3**).

Moreover, 61 patients (19%) received amino acid supplementation (Aminess N®; Recip AB, Stockholm, Sweden, containing per tablet: 45 mg histidine, 135 mg valine, 60 mg isoleucine, 90 mg leucine, 65 mg lysine, 90 mg methionine, 70 mg phenylalanine, 65 mg threonine, 25 mg tryptophan, and 75 mg tyrosine), However, the concentrations of total and individual BCAAs did not differ significantly between those who received amino acid supplements and those who did not (**Table S3**).

Patients with protein–energy wasting (PEW) had significantly lower total BCAA concentrations than well-nourished CKD patients (254 [224–305] µmol/L vs. 290 [237–334] µmol/L, P = 0.002; **Fig. S4**). In addition, TNF-α concentrations were significantly higher in patients with PEW compared with those with normal nutritional status (11.1 [8.5–15.2] pg/mL vs. 9.8 [7.9–12.5] pg/mL, P = 0.03; **Fig. S5**).

**Table S1**. Comparison between total (T-BCAA) and individual branched-chain amino acids concentrations in 83 controls (C) and 328 CKD stage 5 patients.

|  | **C** | **CKD 5** | **P-value** |
| --- | --- | --- | --- |
| **T-BCAA,** μmol/L | 392 (343-459) | 281 (232-329) | <0.0001 |
| **Valine,** μmol/L | 219 (190-250) | 152 (126-179) | <0.0001 |
| **Leucine,** μmol/L | 116 (101-138) | 67 (54-84) | <0.0001 |
| **Isoleucine,** μmol/L | 58 (50-70) | 56 (43-68) | 0.09 |

Data presented as median (IQR, interquartile range). Clinical characteristics of controls are described in Mukai H et al PMID: 29702682, and Ghanavatian S et al PMID: 23567479

**Table S2.** Comparison of individual branched-chain amino acid (BCAA) concentrations (μmol/L) in 328 CKD stage 5 patients, analyzed by sex, presence of diabetes mellitus (DM), cardiovascular disease (CVD), and statin use, in relation to their respective counterparts.

|  |  | **Valine** | **P-value** | **Isoleucine** | **P-value** | **Leucine** | **P-value** |
| --- | --- | --- | --- | --- | --- | --- | --- |
| **Sex** | Males | 158 (130 - 191) | <0.0001 | 60 (50 – 72) | <0.0001 | 71 (58 – 88) | <0.001 |
|  | Females | 142 (111 – 165) |  | 47 (39 - 61) |  | 62 (49 – 79) |  |
| **DM** | Yes | 149 (127 - 177) | 0.84 | 56 (42 – 68) | 0.61 | 72 (55 – 89) | 0.08 |
|  | No | 152 (125 - 181) |  | 56 (43 - 69) |  | 65 (53 - 82 |  |
| **CVD** | Yes | 147 (122 – 181) | 0.42 | 54 (43 – 66) | 0.33 | 70 (52 - 87) | 0.59 |
|  | No | 152 (128 - 178) |  | 57 (43 - 70) |  | 67 (55 – 82) |  |
| **Statin** | Yes | 150 (122 - 174) | 0.34 | 55 (41 - 69) | 0.54 | 68 (81 - 85) | 0.88 |
|  | No | 153 (126 - 181) |  | 56 (43 - 68) |  | 67 (54 - 83) |  |

Data presented as median (IQR, interquartile range).

**Table S3**. Comparison of the concentrations of total and individual branched-chain amino acids (BCAAs) between 61 CKD stage 5 patients on amino acid supplementation and 267 patients without supplementation.

|  | **Aminess** | **No Aminess** | **P-value** |
| --- | --- | --- | --- |
| **Number (%)** | 61 (19%) | 267 (81%) |  |
| **BCAAs** | 275 (236-334)) | 283 (230-332) | 0.77 |
| **Valine** | 152 (123-181) | 146 (131-184) | 0.74 |
| **Isoleucine** | 56 (40-71) | 58 (43-69 | 0.95 |
| **Leucine** | 70 (55-85) | 66 (53-82) | 0.51 |

Data presented as median (IQR, interquartile range). Oral amino acid supplementation (Aminess N; Recip AB, Stockholm, Sweden) contains 45 mg histidine, 135 mg valine, 60 mg isoleucine, 90 mg leucine, 65 mg lysine, 90 mg methionine, 70 mg phenylalanine, 65 mg threonine, 25 mg tryptophan, and 75 mg tyrosine.

**Table S4**. Competing-risk regression analysis models for all-cause and cardiovascular 5-year mortality risk based on tertiles of branched-chain amino acids (BCAAs), valine, isoleucine and leucine, respectively, among 328 CKD stage 5 patients.

|  | **All-cause mortality** | | **Cardiovascular mortality** | |
| --- | --- | --- | --- | --- |
|  | **sHR [95% CI]** | **p-value** | **sHR [95% CI]** | **p-value** |
| **BCAAs** |  |  |  |  |
| Low tertile | 1.62 (0.88 - 2.98) | 0.12 | **2.37 (1.08 - 5.21)** | **0.03** |
| Middle tertile | 1.01 (0.56 - 1.83) | 0.96 | 0.81 (0.36 - 1.83) | 0.62 |
| **Valine** |  |  |  |  |
| Low tertile | **2.05 (1.10 – 3.79)** | **0.02** | **2.46 (1.15 – 5.26)** | **0.02** |
| Middle tertile | 1.40 (0.74 – 2.63) | 0.29 | 1.17 (0.51 – 2.69) | 0.69 |
| **Isoleucine** |  |  |  |  |
| Low tertile | 1.20 (0.56 – 2.19) | 0.55 | 1.63 (0.74 – 3.58) | 0.21 |
| Middle tertile | 0.99 (0.57 – 1.73) | 0.96 | 0.97 (0.47 – 1.97) | 0.93 |
| **Leucine** |  |  |  |  |
| Low tertile | 0.66 (0.35 – 1.23) | 0.19 | 0.94 (0.43 – 2.04) | 0.87 |
| Middle tertile | 0.96 (0.56 -1.66) | 0.90 | 1.21 (0.59 – 2.43) | 0.60 |

Values shown are sub-hazard ratio (sHR) with 95% confidence interval (95% CI). The high tertile served as the reference group. Each model was adjusted for age, sex, diabetes mellitus, cardiovascular diseases, estimated glomerular filtration rate, body mass index, serum albumin, atherogenic index of plasma and plasma insulin. Significant values are marked as bold.

**Fig S1.** Participant flow chart of incident dialysis patients in Stockholm with planned start of dialysis who were enrolled in cohort study December 1994-October 2005.

## Enrollment

^a^ Estimation based on source: <https://www.medscinet.net/snr/arsrapporter.aspx>

**Estimated number of patients with planned start of dialysis in catchment area Stockholm region 1994 – 2005 (n≈900)^a^**

Excluded

- Age <18 years
- Age >70 years
- Overt infection or acute vasculitis

Included

- CKD stage 5 (eGFR-epi <15 ml/min)
- Willing to participate
- Age 18-70 years

**Eligible for main cohort study (n=560)**

**Included (n=328)**

♦198 men (60%), median age 54 years, median eGFR 6.3 ml/min/1.73 m^2^

♦ Causes of CKD: diabetic nephropathy (26%), hypertension/renal vascular disease (21%), chronic glomerulonephritis (25%), other causes (28%)

**Excluded (n=232)**

- No analysis of plasma amino acids

## Allocation

♦Follow-up up to 60 months; median 29.4 (range 0.9–60) months

♦ Renal transplantation (n= 166); deaths (n=82); lost to follow-up (n=0)

## Follow-Up

**Statistical analysis**
♦ Fine-Gray competing-risk regression analysis.

♦ Restricted cubic spline curve analysis

## Analysis

**Fig S2.** Box plots of total branched-chain amino acids (BCAA) concentration in 328 CKD stage 5 patients without and with diabetes mellitus (**A**), cardiovascular disease (**B**), statin medication (**C**), and male sex (**D**) compared to their respective counterpart groups.

**A**

**B**

**C**

**D**

P = ns

P = ns

P = ns

P < 0.0001

**Fig S3.** Box plots of total branched-chain amino acids (BCAA) and individual BCAA concentrations in male and female healthy subjects.

**Fig S4.** Box plots of total branched-chain amino acids (BCAA) concentrations in well-nourished and malnourished CKD patients.

**Fig S5.** Comparison of tumor necrosis factor-alfa (TNF- α) concentration in well-nourished and malnourished CKD patients.
